# Could Curcumin Modified Silver Nanoparticles Treat COVID-19?

**DOI:** 10.34172/apb.2022.002

**Published:** 2020-12-05

**Authors:** Siukan Law, Chuiman Lo, Jie Han, Albert Wingnang Leung, Chuanshan Xu

**Affiliations:** ^1^Department of Science, School of Science and Technology, Hong Kong Metropolitan University, Ho Man Tin, Kowloon, Hong Kong, China.; ^2^Department of Chemistry, The Chinese University of Hong Kong, Shatin, New Territories, Hong Kong, China.; ^3^School of Nursing, Tung Wah College, 31 Wylie Road, Ho Man Tin, Kowloon, Hong Kong.; ^4^Key Laboratory of Molecular Target and Clinical Pharmacology, State Key Laboratory of Respiratory Disease, School of Pharmaceutical Sciences & Fifth Affiliated Hospital, Guangzhou Medical University, Guangzhou 511436, China.

## Dear Editor,

Silver nanoparticles (AgNPs) have been widely used for diagnosis and therapy on wound healing, arthritic as well as infectious diseases. The size of AgNPs are extremely small ranged from 1 nm to 100 nm, which provides a large surface area to enhance their solubility, chemical stability, and catalytic activity for its functions.^1^ Anti-viral and anti-bacterial are the two common properties of AgNPs, which are attracting scientists’ investigation.

AgNPs have an anti-viral potential against many types of viruses including human immunodeficiency virus, hepatitis B virus, herpes simplex virus, monkeypox virus, and respiratory syncytial virus.^2^ In 2008, Sun L et al. reported that the respiratory syncytial virus was inhibited by AgNPs capped with the poly(N-vinyl-2-pyrrolidone) (PVP), bovine serum albumin (BSA), and a recombinant F protein from RSV (RF 412). AgNPs could bind to the viral surface, block the interaction with G-protein, and distribute on the viral envelope to suppress its combination.^3^

Morris D et al. also proved that AgNPs had anti-viral and immunomodulatory activities in respiratory syncytial virus (RSV) infection of mice. It inhibited the virus replication and decreased the level of pro-inflammatory cytokines (i.e., IL-1α, IL-6, TNF-α) and pro-inflammatory chemokines (i.e., CCL2, CCL3, CCL5). They showed AgNPs was non-toxic when silver nanoparticles size was within 12 nm and dose below 50 mg/mL.^4^

Besides, AgNPs also have an anti-bacterial potential against methicillin-resistant *Staphylococcus aureus* (MRSA). Ip M et al. identified the release of silver ions in the wound dressings from the AgNPs. Silver ions interacted with the thiol groups of enzymes and proteins of bacteria to affect cell respiration and kill the bacteria.^5^ Another study from Duncan et al. demonstrated that the antibacterial activity of AgNPs on Gram-positive and Gram-negative bacteria. The AgNPs anchored on the bacterial cells to cause lysis and release of the silver ion into the cytoplasm to inhibit an electron transport chain in the cell, inactivate the bacterial protein synthesis, inhibit the DNA replication, and activate the generation of reactive oxygen species (ROS) to destroy the membrane protein, subsequently resulting in the bacterial cell death.^6^

Recently, Zachar O et al. reported that AgNPs were effective in the prevention and treatment of COVID-19. The concentration of AgNPs colloids are around 10 μg/mL and the particle sizes between 3 nm to 7 nm. However, these formulations were only investigated at the early stages for COVID-19. It’s required to work more before going on to the next stages.^7^ Basically, the pathogenesis of COVID-19 is caused by a SARS-CoV-2 virus. It binds to epithelial cells in the nasal cavity via the angiotensin-converting enzyme 2 (ACE-2) receptor. The SARS-CoV-2 virus migrates down the respiratory tract to induce infection in the airway and lung, causing damage, apoptosis of alveoli cells, and finally, lead to lung inflammation. If the continuous self-replication of SARS-CoV-2 virus in the tissues, it results in more severe scarring and fibrosis in the lung.^8^

Nowadays, traditional Chinese herb is widely used within the COVID-19 outbreak such as “Curcumin”. It possesses anti-viral and anti-bacterial properties. However, curcumin is lower solubility and absorption in the human body. AgNPs are the best tools for curcumin to improve its limitations and enhance the original function. A proposed pharmacological mechanism of AgNPs encapsulated with curcumin is the same as AgNPs itself. Curcumin blocks the active site of ACE-2 and attaches to the viral envelope lead to inhibit its replication, prevent the infection of the respiratory tract.

In the past, it had several shreds of evidence about AgNPs encapsulating curcumin with increased anti-viral and anti-bacterial activities. Yang XX et al. discovered that curcumin acted as reducing and capping agents in the synthetic route of AgNPs. AgNPs modified by curcumin with a stronger anti-viral property and higher efficient inhibition effect against respiratory syncytial virus (RSV) infection.^9^ Gupta A et al. also developed curcumin-cyclodextrins loaded into bacterial cellulose-base for wound dressing applications. These silver nanoparticle-loaded cellulose hydrogels had a strong anti-bacterial property with high cytocompatibility. It could inhibit three of the common wound-infecting pathogenic microbes including *Staphylococcus aureus*, *Pseudomonas aeruginosa*, and *Candida auris*.^10^

All of the above information demonstrates that AgNPs or curcumin-loaded AgNPs might have potential in treating COVID-19. However, much more work needs to be done such as basic cell culture, cytotoxicity, animal models, clinical trials, and safety assessments in the human body.

## Ethical Issues

Not applicable.

## Conflict of Interest

Authors declare no conflict of interest in this study.
